# Lymphocytic Lymphoma Transforming into Hodgkin Lymphoma in Sub-Saharan Africa: Case Report and Literature Review

**DOI:** 10.3390/hematolrep16030050

**Published:** 2024-08-05

**Authors:** Sokhna Aïssatou Touré, Dibor Niang, Serigne Mourtalla Gueye, Mohamed Keita, Alioune Badara Diallo, Elimane Seydi Bousso, Fatma Dieng, Blaise Felix Faye, Moussa Seck, Saliou Diop

**Affiliations:** 1 Hematology Department, Cheikh Anta Diop University, Dakar 10700, Senegal; 2 Pathology Department, Gaston Berger University, Saint-Louis 234, Senegal; 3 Clinical Hematology Unit, National Blood Transfusion Center, Dakar 5002, Senegal

**Keywords:** Richter, Hodgkin, lymphocytic lymphoma, Sub-Saharan Africa

## Abstract

The Hodgkin variant Richter syndrome (HvRS) is an infrequent complication occurring in 1% of lymphocytic lymphoma/chronic lymphocytic leukemia patients. We report a case of HvRS diagnosed in Sub-Saharan Africa. A 63-year-old patient was consulted for the investigation of an abdominal mass that had been evolving for 5 years prior to admission. His history revealed night sweats, 13% weight loss in 3 months and persistent pruritis. Examination revealed bilateral cervical axillary and inguinal macroadenopathies, painless abdominal distension, pruritic lesions and WHO 2 PS. The blood count showed anemia at 9.5 g/dL. Histology revealed a lymphomatous proliferation of diffuse architecture, nodular in places, with Hodgkin and Sternberg cells associated with small lymphocytes, histiocytes and eosinophilic polymorphs. Immunohistochemistry showed CD20, PAX5, BCL2, CD5, CD23 and MYC positivity; Ki67 at 10% and cyclin D1, BCL6 and CD10 negativity; CD30 positivity on Hodgkin and Sternberg cells that remained CD20 negative; difficulty interpreting CD15; EBV positivity (EBERs); and CD3 and CD5 positivity on reactive T cells. CD138 and kappa and lambda light chains were non-contributory. The extension work-up classified the patient as Ann Arbor stage III B with a Hasenclever score of 3/7. This case illustrates the difficulties in diagnosing HvRS in our countries, where the number of haematopathologists is insufficient and the technical facilities are limited.

## 1. Introduction

Richter syndrome (RS), or Richter transformation, is the occurrence of an aggressive lymphoma in a patient with previously discovered or concomitant chronic lymphocytic leukemia (CLL) or lymphocytic lymphoma (LL) [1]. 

It is reported in 2–10% of CLL/LL patients, with an incidence rate of 0.5–1% per year, and can develop in treatment-naive patients, but is more common after therapy [2].

CLL most often progresses to diffuse large B-cell lymphoma (95–99%) and rarely to Hodgkin lymphoma (1–5%), also known as Hodgkin variant Richter syndrome (HvRS) [3,4]. HvRS is a very infrequent form with a poorly understood pathophysiology and a poor prognosis. Although HvRS has a worse prognosis than de novo classical HL, it has a better prognosis than RS with the DLBCL variant. It is manifested by a change in the histopathological and biological characteristics of the original clone [3,5]. Hodgkin lymphoma is thought to arise from B cells of the germinal or post-germinal center, with evidence of somatic hypermutation V (H). Given the histogenesis of CLL/LL and Hodgkin lymphoma, only CLL/LL derived from V(H)-mutated B cells is hypothesized to progress to the Hodgkin lymphoma variant of Richter syndrome [6]. At a molecular level, the presence of some genomic markers increases the risk of Richter transformation, including unmutated *IGHV* status, *IGHV* stereotyped subset number 8 (IGHV4-39-IGHJ5), activating *NOTCH1* mutations, *TP53* deletion and/or mutation, and del11q [4].

HvRS is usually treated like de novo classical HL using ABVD or BV-AVD regimens. PD-1 inhibition has shown good results in patients with relapsed CHL-RS. Also, patients who had not previously received purine analogues were shown to have better survival than those who had already received a purine analogue [4].

In the literature, we found case series of reported HvRS, the majority of which were reported in CLL patients undergoing treatment [7]. In Africa, the few cases reported do not specify Richter type [8]. 

In Senegal, the prevalence of CLL/LL is estimated to be 11.2 cases per year, but no case of transformation into Richter syndrome has been reported to date. We therefore report the first case of concomitantly discovered HvRS diagnosed in Senegal.

## 2. Material and Methods

The patient data were collected from their medical records. The blood count was performed using the Sysmex XN1000 automated system (Kobe, Japan). CT scans were performed on a Siemens 16-slice, 32-slice machine (Munich, Germany).

Immunohistochemistry was performed by a manual method as follows:

- Slides were prepared by cutting the blocks with a microtome into 4-micron strips, then spread out on super-adhesive slides incubated at 37° for 1 h.
- Dewaxing of preparations was performed by successive immersion in solvents (xylene 2, absolute ethanol, ethanol 80%, ethanol 70%, tap water and distilled water).
- Heat unmasking was performed by immersing the preparations in a citrate buffer of pH 6.1 (K802321-2 DAKO, Agilent, Santa Clara, CA, USA). After cooling to room temperature, the slides were immersed in distilled water.
- The slides were rinsed and marked by placing them in the wet chamber and rinsing them once with PBS buffer. The slides were drained and dried before being marked with a hydrophobic marker (PAPEN).
- Epitopes were blocked by immersing the slides in a solution of hydrogen peroxide (H_2_O_2_). Rinsing was performed using PBS 3 times.
- Primary antibodies were applied to the slides and incubated overnight in a humidity chamber. There was no dilution because the antibodies were ready to use. Two control slides were used for each antibody. After incubation, the deposited antibodies were rinsed with PBS buffer 3 times for 5 min.
- The deposition of peroxidase-labelled secondary antibody (K802321-2 DAKO Envision Flex high PH kit) was performed. Incubation lasted 20 min in a humid chamber, followed by rinsing with PBS buffer 3 times for 5 min each.
- Revealing antigen–antibody bonds: chromogen substrate (DAB: 3,3′-diaminobenzidine) was applied to the tissue in the darkroom for 10 min. The slides were rinsed with distilled water for 15 s.
- Counter-staining was performed by staining preparations with a drop of MAYER hematoxylin solution for 30 s, then rinsing them with distilled water before being rehydrated and mounted between slides and coverslips.

The antibodies used were as follows: CD20, PAX5, BCL2, CD5, CD23, Ki67, MYC, Cyclin D1, BCL6, CD10, CD30, CD15, CD3, CD5, CD138, Kappa and Lambda light chains and EBERs probe.

The patient’s free and informed consent was obtained for the scientific use of his medical data. 

## 3. Case Description

This was a 63-year-old patient with no specific pathological history, referred to our department for the investigation of an abdominal mass. The symptoms began 5 years before his admission, with the development of progressive abdominal bloating that subsided after taking vegetable charcoal. A few years later, bilateral inguinal adenopathy associated with weight loss appeared. After 5 years of symptomatic and traditional treatment, he was admitted to our department.

Initial examination revealed bilateral, firm, painless cervical axillary and inguinal macroadenopathies that were mobile in both superficial and deep planes, with no tendency to fistulize or compressive signs. The cervical adenopathies were clearly asymmetric, with the largest diameter measuring 6 cm. Painless abdominal distension was also noted, with no transit disorders. In addition, he reported persistent pruritis, with pruritic lesions objectified on examination of the cutaneous–epidermal system. He also reported night sweats associated with a 13% weight loss over 3 months.

The blood count showed a normal white blood cell count of 4.65 G/L, including 1.56 G/L lymphocytes and 0.02 G/L eosinophils; microcytic hypochromic anemia at 9.5 g/dL; and a platelet count of 377 G/L.

**Figure 1:**
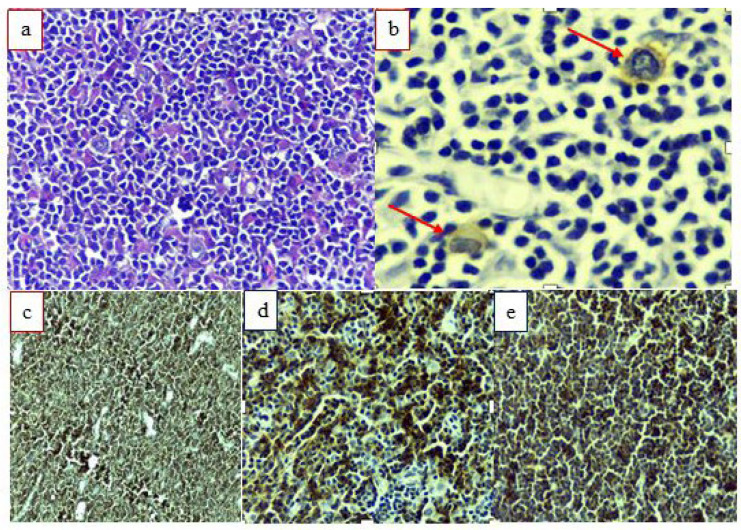
Microphotographs corresponding to the histopathological aspects and molecular profile of RICHTER syndrome. (**a**) diffuse proliferation of small mature lymphomatous cells with hyperchromatic nuclei surrounding the rare Sternberg cells; (**b**) CD30 expression by two Sternberg cells; (**c**–**e**) labeling of the lymphoma population with CD20, CD23 and CD5. Red arrow shows Reed-Sternberg cells.

A histological study of adenopathy showed a lymphomatous proliferation of diffuse architecture that was nodular in places. Small, mature cells with irregular nuclei were present. Attention was drawn to the presence of Hodgkin- and Sternberg-type cells associated with small lymphocytes, histiocytes and eosinophilic polymorphs (Figure 1).

The immunohistochemistry showed positive markers: CD20, PAX5, BCL2, CD5 and CD23. The Ki67 proliferation index showed a clustering of cycling cells illustrating proliferative nests; this was in the order of 10%. MYC also highlighted proliferative nests. Cyclin D1, BCL6 and CD10 were negative. CD30 showed Hodgkin- and Sternberg-like cells, which remained negative for CD20 and were weakly positive for PAX5. CD15 was difficult to interpret and showed rare weakly positive cells (Golgian marking). These were EBV-positive (EBERs), and both CD3 and CD5 showed an enrichment of reactive T cells in these areas. CD138 and the kappa and lambda light chains did not provide any additional information.

Overall, the appearance was that of a lymphocytic-type B lymphoma associated with EBV-positive classical Hodgkin lymphoma.

A cervicothoracic–abdominal–pelvic computed tomography (CT) scan carried out as part of the extension work-up revealed, in addition to superficial adenopathies, posterior mediastinal, retroperitoneal and iliac adenopathies. The latter displaced the bladder posteriorly and sheathed the iliac vessels without compressing them. The rest of the investigations (osteo-medullary biopsy, LDH, viral serology) were unremarkable.

He was classified as stage III B according to the Ann Arbor classification. The Hasenclever score was used to assess our patient’s prognosis; he had 3/7 poor prognostic factors (hemoglobin < 10.5 g/dl, male sex, age > 45 years). Therapeutically, the R-ABVD protocol (rituximab, anthracycline, bleomycin, vincristine, dacarbazine) had been proposed to him following a hematological multidisciplinary consultation meeting. However, he has not been treated to date due to financial problems.

## 4. Discussion

Progression from lymphocytic lymphoma to classical Hodgkin lymphoma as a form of Richter transformation is an infrequent but well-documented event that occurs in around 1% of patients with lymphocytic lymphoma (LL)/chronic lymphocytic leukemia (CLL) [9]. A Mayo Clinic study shows that the 10-year incidence of Hodgkin lymphoma (HL) in newly diagnosed CLL/LL patients is around 0.5% (1/200 CLL patients) [7]. Similarly, several studies have reported that HL develops in around 0.1–0.4% of CLL/LL patients at long-term follow-up [2,10,11]. However, it should also be considered that there is a close overlap in clinical signs between lymphocytic lymphoma and Hodgkin transformation, and that the diagnosis of Richter syndrome requires biopsy.

It is highly likely that many cases of transformation have been missed and classified as refractory forms of CLL/LL in Sub-Saharan African countries. 

The case of our 63-year-old patient is the first to be reported from Sub-Saharan Africa, and his symptomatology began 5 years before his consultation in our department. This suggests that our patient had presented with an undiagnosed lymphocytic lymphoma for 5 years. This hypothesis is in line with some studies which maintain that the median time to the development of HL is between 2.6 and 4.6 years after diagnosis of CLL/LL [2,11,12].

Although our patient was diagnosed at the stage of transformation into Hodgkin lymphoma, it is important to remember that Richter syndrome should be strongly suspected in any patient with CLL/LL who presents the following clinico-biological manifestations: “B symptoms” (weight loss, night sweats and fever), a rapid increase in the lymph node volume in a particular territory or an increase in spleen size, hypercalcemia, and an increase in blood lactate dehydrogenase (LDH) levels [13]. Nevertheless, histopathological examination remains the gold standard for diagnostic confirmation, showing the presence of large cells with morphological and immunophenotypic characteristics of Hodgkin–Reed–Sternberg (H-RS) cells, staining positive for CD30 [14,15]. 

Two main histopathological and clinical categories can be distinguished. The first is characterized by the presence of scattered H-RS cells in a typical lymphocytic lymphoma setting, while the second type is identified as a true transformation of lymphocytic lymphoma into classical Hodgkin lymphoma, where the H-RS cells are surrounded by an inflammatory milieu of lymphocytes, histiocytes, eosinophils and plasma cells [16,17]. The latter was found in our patient with CD30+ Reed Sternberg cells on a polymorphic inflammatory background. In our patient, we observed the presence of EBV LMP1, carrying strong transforming and anti-apoptotic potential, and EBER, a profile characteristic of latent EBV type II infection [18]. EBV can be considered a prototype for oncogenic viruses that behave as direct transforming agents. It can infect LL cells, leading to the non-pathogenic latent infection of memory B lymphocytes. The presence of EBV genomes and the consistent expression of viral proteins in all neoplastic cells suggest that tumors develop from a single EBV-infected cell and support its involvement in the pathogenesis of several EBV-related tumors, including HL, and other tumors where a causal role for EBV seems unlikely, e.g., CLL/LL [19,20]. In general, type II transformation has a poor prognosis. Previous studies have shown that the median survival of patients with Hodgkin transformation ranges from 4 to 40 months [2,3,7,11,12]. 

We know that there are some high-risk genomic characteristics of CLL that can increase the risk of transformation to RS [4]. Unfortunately, these genetic and molecular anomalies were not investigated in our patient. 

Our patient is not yet on treatment, but we have proposed to him the R-ABVD protocol.

Our patient had a very low socio-economic status. In fact, in our countries with limited resources, there is no health insurance system for all patients. This means that patients have to pay for everything themselves, including medical tests, hospitalization and the acquisition of drugs for treatment. Nevertheless, the government, through its social policy, is trying to subsidize certain oncological drugs.

The most effective chemotherapy regimen for CLL/LL transformation has not been established. These patients most often receive combination chemotherapy targeting HL, including MOPP, ABVD and CVPP, with or without radiotherapy. In some cases, other aggressive cytotoxic regimens with or without rituximab have been used [21]. In general, the disease response and clinical outcome are poorer than in de novo HL [9]. The overall response rate is <50%, and overall patient survival is short (in one cohort analyzed, it was around 0.8 years, and in another, 1.7 years) [11,12,17]. However, PD-1 inhibition has shown good results in patients with relapsed CHL-RS [4]. 

## 5. Conclusions

We report here an infrequent case of HvRS with considerable diagnostic delay. This shows the importance of considering Richter syndrome in the differential diagnosis of patients with CLL/LL, especially those presenting with signs of B-progression (unexplained fever, weight loss, sweating). This case also illustrates the difficulty of establishing this diagnosis, especially in countries where the number of haematopathologists is insufficient and the technical facilities are very limited.

## Data Availability

Data are available from the corresponding authors.
